# The RNAsnp web server: predicting SNP effects on local RNA secondary structure

**DOI:** 10.1093/nar/gkt291

**Published:** 2013-04-27

**Authors:** Radhakrishnan Sabarinathan, Hakim Tafer, Stefan E. Seemann, Ivo L. Hofacker, Peter F. Stadler, Jan Gorodkin

**Affiliations:** ^1^Center for non-coding RNA in Technology and Health, University of Copenhagen, Grønnegårdsvej 3, 1870 Frederiksberg C, Denmark, ^2^Department of Veterinary Clinical and Animal Sciences, University of Copenhagen, Grønnegårdsvej 3, 1870 Frederiksberg C, Denmark, ^3^Bioinformatics Group, Department of Computer Science, and Interdisciplinary Center for Bioinformatics, University of Leipzig, Härtelstraße 16-18, D-04107 Leipzig, Germany, ^4^Department of Theoretical Chemistry, University of Vienna, Währingerstraße 17, A-1090 Wien, Austria, ^5^Faculty of Computer Science, Bioinformatics and Computational Biology group, University of Vienna, Währingerstraße 17, A-1090 Wien, Austria, ^6^Max Planck Institute for Mathematics in the Sciences, Inselstraße 22, D-04103 Leipzig, Germany, ^7^RNomics Group, Fraunhofer Institut für Zelltherapie und Immunologie – IZI Perlickstraße 1, D-04103 Leipzig, Germany and ^8^Santa Fe Institute, 1399 Hyde Park Rd, Santa Fe, NM 87501, USA

## Abstract

The function of many non-coding RNA genes and *cis*-regulatory elements of messenger RNA largely depends on the structure, which is in turn determined by their sequence. Single nucleotide polymorphisms (SNPs) and other mutations may disrupt the RNA structure, interfere with the molecular function and hence cause a phenotypic effect. RNAsnp is an efficient method to predict the effect of SNPs on local RNA secondary structure based on the RNA folding algorithms implemented in the Vienna RNA package. The SNP effects are quantified in terms of empirical *P*-values, which, for computational efficiency, are derived from extensive pre-computed tables of distributions of substitution effects as a function of gene length and GC content. Here, we present a web service that not only provides an interface for RNAsnp but also features a graphical output representation. In addition, the web server is connected to a local mirror of the UCSC genome browser database that enables the users to select the genomic sequences for analysis and visualize the results directly in the UCSC genome browser. The RNAsnp web server is freely available at: http://rth.dk/resources/rnasnp/.

## INTRODUCTION

Characteristics of the 3D structure are essential for the proper functioning of many non-coding RNAs and *cis*-acting regulatory elements of messenger RNAs (mRNAs). These structural RNAs exhibit their function through binding to proteins, other RNA molecules or metabolites. For example, the binding of the *cis*-acting iron-responsive element (IRE) with IRE-binding proteins regulates the Ferritin light chain (*FTL*) gene expression to maintain iron homeostasis in vertebrates (1). The occurrence of nucleotide variant as single nucleotide polymorphisms (SNPs) or mutations in these RNA sequences can alter their structure and affect the molecular function. A well-known example is the mitochondrial transfer RNA (tRNA) mutations that disrupt the structure of tRNA and cause tRNA dysfunction, leading to a variety of severe diseases (2). Similarly, SNPs in both coding (3) and non-coding regions of mRNAs are known to affect the structure and stability of mRNAs that causes aberrant gene regulation [reviewed in (4)]. In particular, SNPs in and around microRNA target sites within the untranslated regions (UTR) of mRNAs have been shown to affect microRNA-mediated regulatory function owing to the structural change induced by the SNPs (5,6). In contrast to natural variants such as polymorphisms or rare mutations, the point mutations introduced by mutagenesis experiments have been widely used to study the sequence-structure relationship of RNAs [e.g. (7)].

RNA folding is a hierarchical process in which the primary RNA sequence initially folds back onto itself to form secondary structure elements, e.g. stems and loops, which are the thermodynamically most favorable configurations. Further, the interactions between these secondary structural elements in 3D space add additional (long-distance) base pairs forming the RNA tertiary structure (8). As the secondary structure is assumed to fold before any tertiary interactions, secondary structure can be predicted independently of tertiary structure information. RNA folding algorithms such as RNAfold (9) and Mfold (10) use dynamic programming to predict the thermodynamically most stable secondary structure by minimizing the free energy of the molecule. Using these RNA folding algorithms, the structural impact of SNP or mutation on the RNA sequence can be predicted by comparing the secondary structures of wild-type and mutant (containing SNP or mutation) RNA sequences. The structural impact can be differentiated as local or global with respect to changes in base pairs at the local or global RNA secondary structure (11,12).

In recent years, web servers such as RDMAS (13), RNAmutant (14), SNPfold (12), RNAmute (15) and corRna (16) have been developed to predict the effect of single or multiple-point mutations on the RNA secondary structure. However, these web servers use global folding method to predict the minimum free energy (MFE) structure or ensemble of secondary structures. Hence, for long RNA sequences, the global folding methods require large computational resources. Moreover, prediction accuracy of RNA folding algorithms deteriorates with increasing sequence length. Those approaches further use global metrics to characterize SNP disruptiveness, resulting both in a lack of sensitivity for SNPs disrupting local self-contained structures and in an inability to localize the region impacted by the SNPs. Thus, RNAsnp (17) has been developed to aid the prediction of SNP-induced structural changes in local regions of RNA secondary structure. RNAsnp features both global and local folding methods to compute the ensemble of secondary structures. Furthermore, we have shown that the prediction of SNP effects using the ensemble of secondary structures provides more information than comparing only MFE structures (17). Nevertheless, the prediction of SNP effects on RNA structures remains a difficult challenge: a recent benchmark (18) showed that the agreement of computational assessments agrees much less than perfectly with SHAPE-based measurements of structural effects.

The web server based on RNAsnp provides a convenient interface to provide input data to RNAsnp and to select different modes of operation. It helps visualize the output using informative graphical representation, such as dot plot matrices comparing pair probabilities for wild-type and mutant. In addition, the web server is connected to a local mirror of the University of California, Santa Cruz (UCSC) genome browser database (19) that enables the users to select the genomic sequences of interest for analysis and to visualize the results in the UCSC genome browser (http://genome.ucsc.edu/).

## ALGORITHM

The details of the RNAsnp algorithm have previously been described in (17). Here, we summarize the methodology of RNAsnp and its specific features. RNAsnp is a comprehensive program that has three different modes of operation:

‘Mode 1’ is designed to predict the effect of SNPs on short RNA sequences (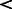
1000 nt), where the base pair probabilities of the wild-type and mutant RNA sequences are calculated using the global folding method RNAfold (9). The structural difference between wild-type and mutant is computed using Euclidean distance or Pearson correlation measure for all sequence intervals (or local regions) using a simple dynamic programming algorithm. Finally, the interval with maximum base pairing distance or minimum correlation coefficient and the corresponding *P*-value is reported.

‘Mode 2’ is designed to predict the effect of SNPs on large RNA sequence. Here, the base pair probabilities are calculated using the local folding method RNAplfold (20). As a first step, the structural difference is calculated using the Euclidean distance measure for all sequence intervals of fixed window length. In the second step, the sequence interval with maximum base pair distance is selected to re-compute the difference for all internal local intervals. The interval with maximum base pair distance and the corresponding *P*-value is reported.

‘Mode 3’, the combination of aforementioned modes 1 and 2 is designed to screen all possible structure-disruptive SNPs in an input sequence using a brute-force approach. First, Mode 2 is applied to evaluate the SNP effect for all possible substitutions at every nucleotide position. Second, the most significant SNPs are subjected to Mode 1 to re-compute the structure effect using a global folding approach. The SNPs that have significant local structural effect are finally reported.

## WEB SERVER

### Usage

The web server offers a common input page to operate the three different modes of RNAsnp. For Modes 1 and 2, the required inputs are a single RNA sequence in FASTA format and one or more SNPs or mutants whose structural effect needs to be predicted. In case of Mode 3, only the RNA sequence is required as input. The input sequence can be either entered in the text box or uploaded as a text file or selected from the local UCSC genome database. To make the computational source available for all users, the upper limit on the RNA sequence length for Mode 3 is limited to 1000 nt. The input SNPs can be either entered in the text box or uploaded as a text file. By default RNAsnp selects a region of 200 nt directly upstream and downstream of the SNP position, to compute the base pairing probabilities. The default value 200 can be varied between 200 and 800 (inclusive) in multiples of 50 using the drop-down option provided in the ‘Folding window’ section. In case of Mode 1, this value can be changed between 100 and 800. By default, the web server runs in Mode 1 to predict the effect of user-supplied SNPs. The additional parameters associated with each mode are displayed on the selection of mode type. The default values assigned for these parameters are the best values that we found from the application of RNAsnp to known data sets (17). Optionally, the user can assign a job name for each submission and request for an email notification. Detailed description of the input formats and sample files are provided on the website.

After the submission, the web server displays the status of the job along with a unique job ID number. This page will refresh automatically for every 10 s and redirects to the result page once the job is completed successfully. The results are stored on the server for 30 days from the date of submission, and it can be accessed using the job ID.

**Figure 1.**
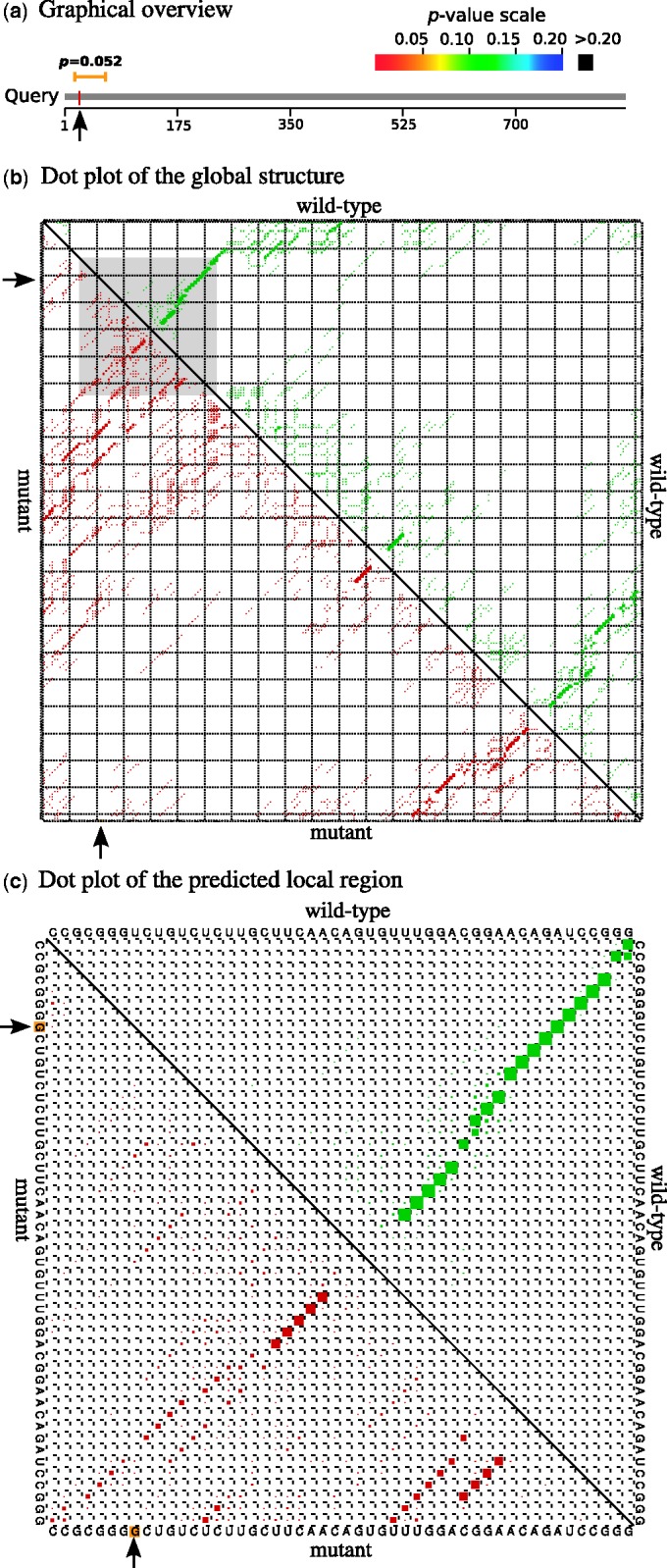
Graphical outputs of RNAsnp web server (Mode 1) generated for the SNP U22G present in 5′ UTR region of the FTL mRNA. (**a**) The graphical overview represents the location of the predicted local region over the query sequence. (**b**) The dot plot shows the global base pair probabilities of the ensemble structures predicted for the region around the SNP position (1–222). The local region predicted with maximum difference is highlighted in gray. (**c**) Base pair probabilities corresponding to the local region (15–64) extracted from (b). In both (b) and (c), the upper triangle represents the base pair probabilities for the wild-type (green) and the lower triangle for the mutant (red). The arrow mark indicates the SNP position.

The result page displays the output under three different sections: ‘Graphic summary’, ‘Description’ and ‘Structure details’. An example of the RNAsnp output for a SNP U22G in the 5′ UTR region of the FTL mRNA is shown in Figure 1. The ‘Graphic summary’ section provides an overview of the location of the disrupted local region in the given input sequence. Two different lines are used to represent the local region and the query sequence. The line representing the local region is colored according to the *P*-value color scale (Figure 1a). Further, the ‘Description’ section provides a detailed output of RNAsnp in tabular format, such as the SNP position, region considered for predicting the base pair probabilities, predicted local region and its corresponding distance or correlation coefficient score along with the significance value. If the input sequence is selected from the local UCSC genome database, a link to visualize the output in UCSC genome browser is provided here. At last, the ‘Structure details’ section displays the secondary structure details of wild-type and mutant RNAs. The base pair probabilities of the ensemble structures of wild-type and mutant RNA sequences are displayed in a dot plot matrix. The indices (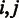) of the matrix show a dot if the bases at position *i* and *j* form a base pair. The size of the dots is proportional to the base pairing probability where small dots indicate low and large dots indicate high probability to form a base pair (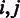). The upper triangle of the dot plot contains the base pair probabilities for the wild-type sequence and the lower triangle for the mutant sequence. The respective wild-type and mutant primary sequences are displayed on the sides of the triangle. For the mutant sequence, the SNP position is highlighted with a yellow box. Figure 1b shows the dot plot of the global structure calculated using RNAsnp Mode 1, where the local region predicted with maximum difference between wild-type and mutant is highlighted in gray. The dot plot matrix corresponding to this local region is separately shown in Figure 1c. For an intuitive way of representing the structural dissimilarity, the global MFE structures of the wild-type and mutant are displayed in planar graph representation. The output shown under each section can be downloaded either individually or in group as a single archive.

### Implementation

The web server is implemented in PHP, HTML and Javascript and runs under Apache. The input data are passed to RNAsnp, and once the job is completed successfully, the user is forwarded to the output page. The figure representing the graphical overview is drawn using the *TikZ* and *PGF* packages (21), whereas the dot plots are generated using the Vienna RNA package (9,22) and post-processed using custom scripts. The *dpzoom.pl* script from Vienna RNA package is used to extract the base pair probabilities of the local region from global base pairing probability matrix. The planar graph representation of secondary structures is drawn using Visualization Applet for RNA (VARNA) (23).

## RESULTS AND DISCUSSION

In (17), the efficiency of RNAsnp was evaluated using a data set SNPs with experimentally confirmed structural effects. Here, we showed the function of RNAsnp web server with the help of two SNPs (U22G and U22G-G14C), whose effect on RNA secondary structure has been studied recently (24). These two SNPs are present in the 5′ UTR region of FTL mRNA. The 5′ UTR region of this mRNA contains a functional RNA element, IRE, which plays a major regulatory role in the FTL mRNA translation (1). The SNP U22G has been reported to disrupt the IRE-structure, whereas the U22G-G14C restores the structure of the mutant IRE to wild-type (24). The FTL mRNA sequence (NM_000146.3) and these two SNPs were given as input to RNAsnp web server to predict the structural effect using Mode 1. The RNAsnp predicted correctly that the SNP U22G has significant local structural effect (*P* = 0.0518), whereas U22G-G14C has no significant local structural effect (*P* = 0.3464). Figure 1 shows the graphical overview and dot plot output for U22G, whereas Figure 1a represents the local region predicted with maximum structural change, which is overlapping with the SNP position. From Figure 1b, it can be seen that the base pair probabilities are mostly similar for the wild-type and mutant sequences, on the exception of the local region predicted by RNAsnp (highlighted in gray). Finally, Figure 1c represents a magnified view of the dot plot corresponding to the structurally disrupted region. The wild-type sequence forms a stem-loop structure, which is, however, disrupted due to the nucleotide change from U to G at position 22 in the mutant sequence. Similar findings were obtained while using RNAsnp Mode 2. These results indicate that the structural disruption of IRE might potentially affect the function of IRE and thus leads to aberrant FTL gene regulation.

**Figure 2.**
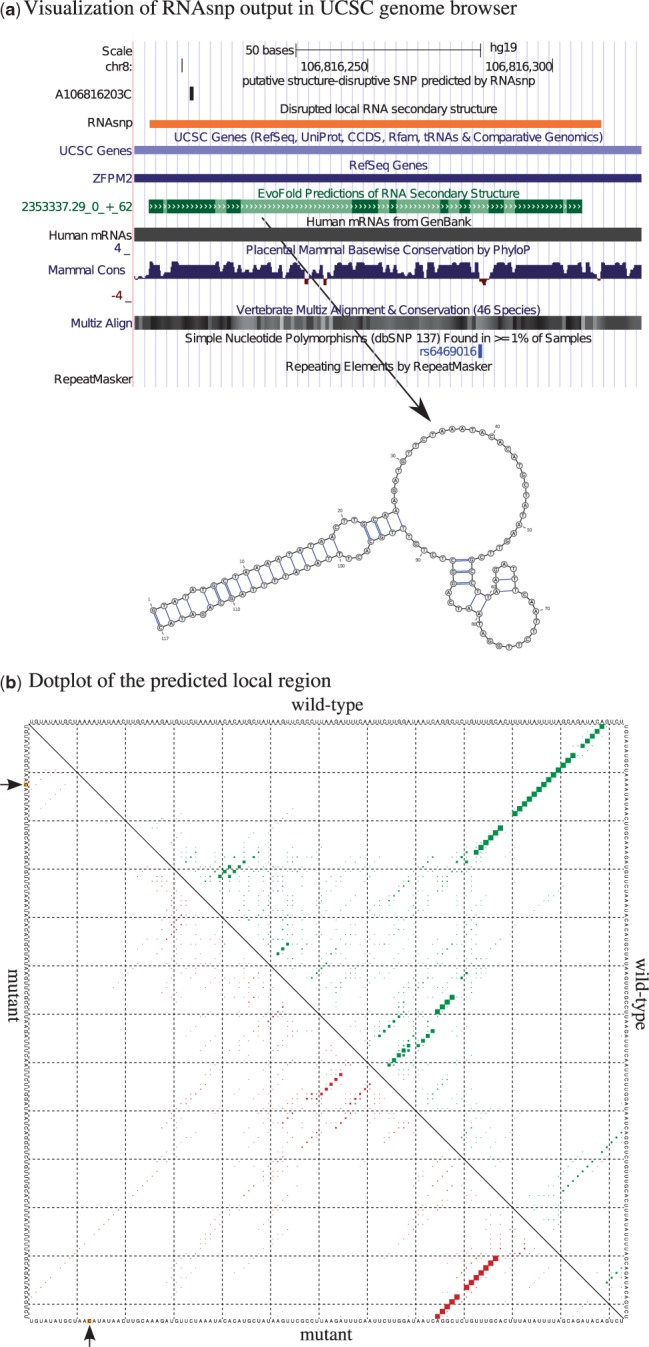
The RNAsnp web server output for a putative structurally disruptive SNP, A106816203C, screened from chromosome 8 of human hg19 assembly. (**a**) The output of A106816203C displayed at the UCSC genome browser. The local region predicted by RNAsnp overlaps with the conserved RNA secondary structure predicted by EvoFold. (**b**) Dot plot shows the base pair probabilities of the local RNA secondary structure, where the upper triangle represents the probabilities for wild-type (green) and the lower triangle represents the mutant (red). The arrow mark indicates the SNP position.

To demonstrate the ability of the RNAsnp web server to search for structure-disruptive SNPs, a genomic region (chr8:106815766-106816766) of length 1000 nt from the human genome hg19 assembly was selected and passed to RNAsnp Mode 3. The web server took ∼20 min for the computation and reported 50 putative structure-disruptive SNPs with *P*-value <0.05. The number of predicted SNPs is reasonably close to the expected 5% at our chosen level of significance. The output provides a link to the UCSC genome browser to get an overview of the RNAsnp output with other information available in the UCSC genome browser. As an example, Figure 2a shows the output for one of the structure-disruptive SNPs, A106816203C, visualized in the UCSC genome browser. The first two tracks are RNAsnp specific and represent the chromosomal location of the structure-disruptive SNP (*P* = 0.0336) and the local region where the maximum structural change was observed due to this SNP. Interestingly, the local region predicted by RNAsnp overlaps with a conserved RNA secondary structure predicted by EvoFold (25) (track highlighted in green color).

Moreover, the base pair probabilities of ensemble structures predicted for the wild-type sequence in RNAsnp (Figure 2a) are favorable for the conformation of the secondary structure predicted by EvoFold (Figure 2a). However, the SNP A106816203C screened by RNAsnp could potential disrupt the RNA secondary structure (Figure 2b) at the conserved region. In addition, we expect that this SNP could be a rare variant because the nucleotide conservation at the SNP position is high with respect to the phyloP score (highlighted in blue histogram), and there is no common sequence variation at this position according to the dbSNP track.

## CONCLUSION

The web server presented here provides a convenient access to the RNAsnp program. The results are shown in a more intuitive graphical manner. In contrast to other web servers with similar functions, the RNAsnp web server can predict local structure changes and report the exact location of the disrupted region and the significance of the structural change in the form of an empirical *P*-value. The RNAsnp output is linked to the different genome annotation track found in the UCSC genome browser, greatly simplifying contextual analysis of the SNP effects. Finally, the web server can help researchers predict the structural effect of natural variants and to screen putative structure-disruptive nucleotide variants for mutagenesis experiments.

## FUNDING

Danish Center for Scientific Computing (DCSC, DeiC); Danish Council for Strategic Research (Programme Commission on Strategic Growth Technologies); Danish Council for Independent Research (Technology and Production Sciences); European Community Seventh Framework Programme [proj. no. 222664, QUANTOMICS]. Funding for open access charge: University of Copenhagen.

*Conflict of interest statement*. None declared.
